# The A.BA.CO. Project and Efforts to Optimize Access to the Sounds of Learning

**DOI:** 10.3390/audiolres15040092

**Published:** 2025-07-25

**Authors:** Eva Orzan, Valeria Gambacorta, Giampietro Ricci

**Affiliations:** 1 Audiology and Otorhinolaryngology Unit, Institute for Maternal and Child Health IRCCS “Burlo Garofolo”, 34137 Trieste, Italy; eva.orzan@burlo.trieste.it; 2 Department of Medicine & Surgery, Section of Otorhinolaryngology, University of Perugia, 06126 Perugia, Italy

**Keywords:** acoustic, learning, communication, school, technologies

## Abstract

**Background/Objectives**: Despite its significant impact on learning, classroom acoustics and students’ hearing difficulties are often overlooked compared with more visible issues like lighting. Hearing loss—frequently underestimated and invisible—affects both students and teachers, potentially leading to fatigue, reduced participation, and academic challenges. The A.BA.CO. project in Italy was developed to address these issues by promoting improved classroom design, technological solutions, and better auditory communication accessibility in schools. **Objective**: This article presents the A.BA.CO. project, offering context and an overview of the preliminary analyses conducted by its multidisciplinary team. The goal is to share insights and propose organizational frameworks, technical solutions, and best practices concerning the hearing, communication, and auditory learning challenges experienced by students with hearing impairments. **Results**: The A.BA.CO. team’s analyses identified key barriers to inclusion for students with (or without) hearing impairments, such as poor classroom acoustics, excessive noise, and suboptimal seating arrangements. The project underscores the importance of improved acoustic environments and the integration of assistive technologies, including speech-to-text systems. The findings highlight the need for interdisciplinary collaboration to design accessible and inclusive educational settings for all learners. **Conclusions**: Embedding educational audiology within school systems—alongside enhancements in classroom acoustics and the use of assistive technologies and other technological solutions—is essential to ensure that all students, regardless of hearing ability, have equitable access to learning and full participation in educational life.

## 1. Introduction

*“When the lights go out in the classroom, no time is wasted getting the bulbs changed, because it is assumed that students cannot learn in school if they cannot see the teacher or their materials due to poor light. If poor acoustics were equally easy to ‘see’ as poor lighting, would we be as diligent in improving the acoustics?”*

[1]

While poor lighting in classrooms is swiftly addressed due to its obvious, visible impact on learning, the less perceptible auditory environment often receives far less attention. If poor acoustics were as immediately noticeable as inadequate lighting, they might elicit the same sense of urgency. Unfortunately, the auditory conditions within classrooms—the acoustic characteristics of the space, the hearing abilities of students and teachers, and the vocal and speech dynamics of educators—are frequently overlooked. Yet, these factors profoundly shape the learning experience and can significantly influence both instructional effectiveness and student achievement [2,3].

Addressing hearing loss in school settings requires a multidimensional perspective. Although many critical aspects of hearing and classroom acoustics are well known within specialized fields, in countries like Italy, they have yet to be considered for their full complexity, interconnection, and significance for transforming educational policies, school and classroom design, and teaching methodologies.

One key issue is the persistent underestimation of how many individuals in schools—students and teachers alike—experience hearing difficulties. Hearing loss is often referred to as an “invisible disability,” and this invisibility can lead to its neglect.

Mackenzie and Smith emphasize that hearing loss is a chronic and frequently unnoticed disability that, if not addressed promptly, can hinder language and cognitive development in children, adversely affecting education, employment, and social interactions [4]. Le Clercq et al. conducted a study revealing that around 15% of children and adolescents in the US, aged 9 to 11, experience some degree of hearing loss, which is frequently mild and unrecognized. Notably, only 10.8% of those with identified hearing loss acknowledged having hearing issues during interviews, suggesting a significant underestimation of the condition by both parents and children [5]. This finding is corroborated by the research conducted by Kenworthy, Klee, and Tharpe, which indicates that school-age children with unilateral hearing loss experience considerable challenges in verbal comprehension relative to their peers with normal hearing, particularly in noisy settings, implying a detrimental effect on academic performance [6]. Savegnago et al. also emphasized that children with hearing loss, including mild or temporary cases, face a heightened risk of learning difficulties and impairments in language comprehension, particularly in school settings characterized by background noise [7].

Moreover, the challenges associated with hearing loss go beyond the act of hearing itself. They can directly affect well-being by increasing listening-related fatigue due to the cognitive and emotional strain of processing speech in challenging environments. Holman et al. (2021) demonstrate that individuals with hearing loss encounter a notable rise in listening fatigue, attributed to the cognitive and emotional demands of language processing in acoustically challenging settings. This fatigue adversely affects psychophysical well-being, both by diminishing energy and motivation directly and by restricting engagement in social and daily activities indirectly [8].

Considering the fluctuations in and interactions among hearing loss, the use of hearing devices, fatigue, and activity levels can significantly impact overall well-being. Children and adolescents with hearing impairments often encounter restrictions in participation such as fewer diverse activities, a reduced intensity of engagement, more time spent at home, and an increased dependence on guardians [9]. These limitations inevitably influence their academic progress and personal development. 

Hearing in schools is perhaps one of the most important, but often least considered, factors when designing a classroom and addressing students’ hearing disabilities [10]. The A.BA.CO. project (Abbattimento delle Barriere Comunicative—Breaking Down Communicative Barriers), promoted by the Friuli Venezia Giulia Region in Italy and supported by the Disability Office of the Italian Council of Ministers, was developed to address several of the critical issues surrounding hearing loss and communication access, particularly within educational and public service contexts. This article describes the A.BA.CO. project and contextualizes and summarizes some of the preliminary work of the multidisciplinary project team, with the aim of sharing knowledge and proposing organizational models, technical solutions, and best practices in the field of hearing, communication, and auditory learning challenges for students with hearing difficulties.

### The A.BA.CO. Project

The A.BA.CO. project (Abbattimento delle barriere communicative; Breaking Down Communicative Barriers) was formally launched on 1 June 2021 and refinanced in 2024. It is the result of a collaboration between the Audiology Department of the Institute for Maternal and Child Health IRCCS Burlo Garofolo—a national center of excellence in maternal and child healthcare in Trieste, Italy—and the ENS (Ente Nazionale Sordi—National Deaf Organization) of the Friuli Venezia Giulia Region, Italy. Additional partners included the University of Perugia, the Otolaryngology Department of Perugia University Hospital, and the association FIADDA Umbria Onlus (Famiglie Italiane Associate per la Difesa dei Diritti degli Audiolesi—Italian Families Associated for the Defense of the Rights of the Hearing Impaired), who represent the families of individuals with hearing impairments.

Together, these institutions worked across regional boundaries (Friuli Venezia Giulia and Umbria regions, Italy) with a shared commitment to promoting educational inclusion, social participation, and communicative equity for deaf and hard-of-hearing individuals of all ages. By addressing both the technological and cultural dimensions of communication barriers, the A.BA.CO. project serves as a model for inclusive policy and practice in education and public service.

The project focuses on promoting communicative autonomy for individuals with hearing loss or deafness by implementing targeted actions and technological innovations. These measures aim to ensure full access to communication and information from the early school years onward, respecting individual communication preferences and needs. In this context, the A.BA.CO. initiative recognizes and integrates the two communicative–linguistic modalities among people with hearing loss—oral language and sign language—structuring the project into three distinct yet complementary branches of action: A.BA.CO. Part I, II, and III (Table 1).

The first and most ambitious component of the A.BA.CO. project (A.BA.CO. Part I) focused on the removal of communicative and comprehension barriers in school settings, with the ultimate goal of fostering autonomy and full inclusion for students with hearing loss or deafness. This phase was coordinated by Dr. Eva Orzan, Director of the Audiology Department at IRCCS Burlo Garofolo, a national center of excellence in maternal and child health. The key initiatives of Part I included the following:

- An in-depth, multidisciplinary analysis of the communication and information access needs of students at both the secondary school level (Friuli Venezia Giulia region, Italy) and the university level (University of Perugia, Umbria region, Italy). This process not only highlighted the diversity of needs among students with hearing impairments but also helped to establish an effective interprofessional network to support future actions.
- The development and testing of an innovative AI-based subtitling system (the A.BA.CO system), capable of synchronizing text with both audio and visual components in real time. This system was designed with flexibility in mind, considering a wide range of hearing impairments and diverse modes of accessing spoken content—whether primarily visual, auditory, or a combination of both. Central to the system’s design was a “design for all” approach, ensuring that students’ individual needs and communication preferences were prioritized, thereby promoting equal opportunities for participation across educational contexts.
- The creation of a comprehensive training resource (A.BA.CO text) for school staff and parents. This document provides essential knowledge on the different manifestations of hearing loss in childhood and adolescence, prosthetic options, rehabilitation methods, reading and writing development, and inclusive teaching practices. To maximize accessibility and dissemination, the training material was also made available in digital format.

Through these actions, A.BA.CO. Part I demonstrated a pioneering model of intervention in educational inclusion, grounded in scientific analysis, technological innovation, and collaborative practice.

To enhance collaboration, streamline project activities, and foster the development of innovative and, above all, user-friendly outcomes for students with hearing loss or deafness, the A.BA.CO. project established an interdisciplinary project team. This team comprised individuals either directly involved in the project or whose interests are positively influenced by its progress and the outcomes of the A.BA.CO. project. The team was intentionally composed of professionals typically engaged in the care, technological support, research, education, and social inclusion of individuals with hearing loss or deafness. These include ENT specialists, neuroscientists, audiologists, speech therapists, psychologists, and educators, alongside students with hearing loss and their families.

The project team convened regularly through online meetings to conduct interdisciplinary analyses and discussions. These sessions covered a broad spectrum of topics, including strategic planning, the assessment of current tools, and the analysis of feedback collected via questionnaires.

## 2. Materials and Methods

The present article provides an introductory overview of a series of scientific articles collected under the A.BA.CO. project (Abbattimento delle Barriere Comunicative—Breaking Down Communicative Barriers). This project report synthesizes and contextualizes a series of independent yet thematically connected investigations, rather than solely presenting original experimental data. The studies were conducted by a multidisciplinary team with the objective of enhancing auditory accessibility and communicative inclusion in educational environments for students with hearing loss.

Each study employed a distinct methodology, including empirical field surveys, acoustic measurements, pilot testing of technological tools, and systematic literature reviews. All studies were coordinated within a unified interdisciplinary framework and directed by the primary objectives of the A.BA.CO. initiative.

## 3. Results

Numerous interdisciplinary meetings and discussions conducted by the multidisciplinary project A.BA.CO. during its initial phases yielded contributions categorized by topic. Nine articles were produced from these contributions, including the current one. Table 2 summarizes the contents of the remaining eight.

The study entitled Hearing and listening difficulties in high schools and universities: the results of an exploratory survey of a large number of students and teachers in the Friuli Venezia Giulia and Umbria regions, Italy by Gambacorta et al. explores how students and teachers perceive hearing and listening in classrooms, based on a survey of 2228 students and 378 teachers from Italian high schools and universities. Hearing difficulties were reported by 8–9% of students. While listening challenges were associated with hearing issues, perceived clarity did not significantly differ between students with or without hearing difficulties. Noise and seating position were key factors affecting listening, varying by education level. Despite existing acoustic standards, many classrooms likely fall short, underscoring the need for improved physical environments and a greater awareness of listening barriers.

Good verbal signals and low background noise are key factors for all children to maximize their understanding of what is being taught. The contribution entitled “Teacher’s vocal effort related to environmental noise in schools” written by Frangipane, Minici, and Chiarella investigated the link between classroom noise levels and teachers’ self-assessed vocal handicap, measured using the Voice Handicap Index (VHI). Conducted with four teachers in an Italian primary school, the study recorded noise levels during lessons and assessed VHI scores afterward. Statistical analyses revealed a significant correlation between noise levels and vocal strain, with higher noise associated with increased VHI scores. Logistic regression showed that noise levels above 59.5 dB were a strong predictor of higher VHI grades. The findings stress the importance of better acoustic management in classrooms to reduce vocal strain and improve teachers’ well-being.

Extra-auditory effects from noise exposure in classrooms, by Asdrubali and colleagues investigated the detrimental effects of noise exposure on both students and teachers in school environments, emphasizing its impact on learning, health, and vocal strain. The study assessed classroom acoustics through both objective measurements of background noise and reverberation, and subjective questionnaires evaluating noise perception, vocal effort, and environmental comfort. Conducted in nine schools across Florence, Perugia, and Rome, the research focused on kindergartens, primary, and secondary schools. The findings revealed moderate noise sensitivity, with major disruptions stemming from classroom chatter, furniture movement, and traffic noise. Only three classrooms met the optimal acoustic standards. The study also found that noise negatively affected students’ concentration, leading to fatigue. Additionally, nearly one-third of teachers reported experiencing high vocal strain during a typical two-hour lesson. The project recommends improving classroom acoustics and implementing training programs for both teachers and students to raise awareness about noise exposure and enhance vocal health and comfort.

Teamwork fosters improved communication, problem-solving, and productivity by combining diverse perspectives and skills. It promotes a positive work environment, ultimately leading to innovation and the development of new ideas. In this perspective, the paper “Speech-to-text Captioning and Subtitling in Schools: The Results of a SWOT Analysis” (strengths, weaknesses, opportunities, and threats) by Fastelli and colleagues examines the implementation of speech-to-text captioning systems in education to support students with hearing loss and overcome communication barriers. Using a SWOT analysis framework, the study involved stakeholders including students, teachers, and audiologists to assess the strengths, weaknesses, opportunities, and threats of adopting such systems. The results show that speech-to-text systems can enhance inclusive education by improving communication and personalized learning. Challenges include cognitive load, synchronization issues, and background noise. The study underscores the importance of user-centered design and interdisciplinary collaboration for optimizing the use of assistive technologies in schools for students with special needs.

The paper Issues and challenges in developing a speech to text system for hearing impaired students—The A.BA.CO. subtitle system story by Onofrei and colleagues traces the multidisciplinary experience in designing and testing a real-time subtitle system that can be both effective and adaptable to the different needs of students with hearing difficulties in the classroom. This paper discusses the system’s design, implementation challenges, and evaluation results. Tested against state-of-the-art transcription models, the system demonstrated similar performance in specific use cases. The project team, comprising diverse professionals, provided valuable insights for system improvements. A summary of suggested enhancements and plans for further evaluation is included. The A.BA.CO. initiative marks a significant advancement in accessibility and inclusion for hearing-impaired students and could serve as a model for similar projects.

The article “Time delay and frequency analysis of remote microphones”, written by Andreatta and colleagues, addresses the increased listening effort and fatigue experienced by children with hearing loss, emphasizing that improved audibility alone (i.e., by hearing aid or cochlear implant) does not reduce the cognitive load in noisy environments of a hearing-impaired student. Therefore, during school activities, it is recommended to use remote microphones that collect the primary signal near the source and transmit it to the aid to be amplified. The A.BA.CO. speech-to-text captioning system for school classrooms would therefore need to use remote microphones to capture the teacher’s speech. However, under this setup, an issue of signal latency arises for students wearing hearing aids or cochlear implants, whose latency is different from that of the remote microphones and may require the development of a temporal coupling solution. The remote microphones combined with two “behind-the-ear” hearing aids, for which transparency was verified, were tested with two different compressions. The findings show a 10–12 ms latency difference between remote microphones and comb filter distortions when remote microphones and hearing aid gains are similar. The communication system will have to foresee different delays based on the model and the brand of remote microphone because similar transmission systems do not have the same time delays. These limits must be considered when estimating the effectiveness of A.BA.CO. system.

Human communication is a core cognitive skill involving dynamic signals like posture, facial expressions, eye and head movements, gestures, and speech. Effective communication in social settings (and therefore also school) relies on the optimal integration of audiovisual speech cues. Although speech comprehension primarily depends on hearing, it is inherently multimodal, combining auditory input with visual cues from facial movements, especially the lips, which move in a coordinated and dynamic way. The review Lip-Reading Revisited: Advances and Unresolved Questions in a Key Communication Skill by Battista et Al explores lip-reading—the ability to understand speech through visual cues—and its significance across the human lifespan. Emerging early in development, lip-reading supports language acquisition and becomes vital in challenging conditions, such as noisy environments or in individuals with hearing or language impairments. The paper integrates findings from psycholinguistics, psychophysics, and neurophysiology to highlight developmental trajectories, individual differences, and cultural influences on this skill. It emphasizes the impact of visual barriers on speech perception and advocates for studying lip-reading in naturalistic contexts. Advancements in analytical tools now enable researchers to better understand the brain’s use of this crucial communicative ability.

For deaf students who use sign language in mainstream education, access to spoken content is vital for inclusion. To support accessibility, classrooms should provide tools like real-time captions, visual aids, notes, and sign language interpreters. Since these students rely heavily on vision, processing simultaneous visual inputs can make it difficult to focus and select relevant information in real time. In this perspective the article On the coexistence of captions and sign language as accessibility solutions in educational settings by Pavani and Leonetti examines the possible coexistence of real-time speech-to-text captions and sign language interpreting to support deaf students who use sign language in mainstream education. A review of empirical studies highlights the effectiveness of captions in improving content access, even when sign language is also used. The findings suggest that integrating both captions and sign language interpreting is feasible and beneficial for deaf signing students. The article also explores recent technological advancements, such as automatic speech-to-text transcription systems and augmented reality tools, and discusses the integration of these solutions within the universal design for learning principles to enhance educational accessibility.

## 4. Discussion

A substantial portion of the A.BA.CO multidisciplinary project team discussions was outlined and summarized in the present work. The main goal of the multidisciplinary A.BA.CO. project team was to provide a critical review of the current communication environments in high schools and universities in order to propose key priorities for future interventions and technological advancements. All findings are intended to serve as a valuable reference for other initiatives aimed at enhancing auditory and communication technologies for students and individuals with hearing impairments. 

The contributions span multiple research questions, methodologies, and outcomes that aim to enhance the accessibility and inclusion of students with hearing impairments in educational settings.

For instance, the project’s survey on hearing difficulties in high schools and universities highlighted significant challenges in classroom acoustics, with noise and seating position being key barriers to effective listening. Hearing in schools is perhaps one of the most important, but often least considered, factors when designing a classroom [10]. The studies on noise exposure and teachers’ vocal strain further emphasize the need for improved acoustic environments to safeguard both students’ learning and teachers’ vocal health.

The project’s exploration of speech-to-text captioning systems and their integration even with sign language also underscores the importance of assistive technologies in fostering inclusive learning. The findings on the performance of speech-to-text systems, including the A.BA.CO. speech-to-text prototype system, suggest that such technologies can significantly improve communication for students with hearing impairments, but customization is crucial to account for technical variations like microphone and hearing aid latency and distortion. All A.BA.CO. outcomes reflect a critical need for interdisciplinary collaboration to optimize the design and implementation of accessibility tools in education, ultimately ensuring that learning environments are conducive to all students, regardless of their auditory capabilities.

There is significant variability in how hearing difficulties among children and adolescents are identified and managed, leading to markedly different auditory learning profiles. While this variability partly reflects the inherent heterogeneity of hearing impairments, it is critically influenced by factors such as the timing of intervention (early or delayed), the type and consistency of treatment, and the quality of follow-up and hearing rehabilitation services [11]. Consequently, students with hearing loss enter school with diverse needs, abilities, and levels of readiness. A primary objective of the A.BA.CO. project is to contribute valuable insights into how well teachers and educational institutions (should) recognize and respond to these differences. 

### Educational Audiology

Alongside the multidisciplinary discussions and team planning, it is worth addressing the necessary support professionals who take responsibility for managing and monitoring good listening in the classroom and effective access to school communication for diverse needs. In Italy, there are figures such as support teachers and communication assistants who are offered in cases of severe hearing disabilities or special needs, but the specific branch specialty recognized internationally as educational audiology has not yet been formally defined or integrated into the school system (see, for example, the educational audiology association www.edaud.org (accessed on 15 April 2025) [12]). In countries where it is established, educational audiology plays a crucial role in supporting academic and social inclusion and—more importantly—the active participation of students with hearing loss. Educational audiology bridges the gap between clinical diagnosis and educational implementation by identifying how hearing impairments affect auditory speech communication, learning, and participation in classroom environments [13]. Educational audiology competence, which includes specialized training in both audiology and educational systems, focuses on the educational and audiological variables of hearing loss in order to design individualized, ongoing support programs. These programs aim to address students’ unique communicative, academic, and psychosocial needs, ensuring equitable access to learning opportunities. The responsibilities would also include referrals for audiological assessments for a wide range of students, including those with multisensory impairments, as well as evaluating speech perception, auditory behaviors, and classroom acoustics. Educational audiology comprises responsibility for the selection, fitting, and monitoring of amplification devices such as hearing aids, cochlear implants, and hearing assistance technologies (e.g., FM systems), ensuring they provide consistent and effective support. The daily troubleshooting and maintenance of these technologies is also a key part of the role. Beyond the technical scope, educational audiologists would collaborate with teachers, parents, and healthcare providers to offer counseling, guidance, and in-service training, raising awareness about the educational implications of hearing loss and providing strategies to enhance teaching practices. The liaisons in making appropriate referrals when other conditions—such as auditory processing disorder or auditory neuropathy spectrum disorder—are suspected also cannot be overlooked [14,15].

Given the significant influence that the auditory learning experience has on both teaching effectiveness and student achievement, support figures that integrate the expertise of school audiology also take on the role of proactive design with the aim of eliminating unnecessary hearing and communication barriers for all students and teachers. They should be tasked with conducting detailed classroom observations to identify potential barriers to listening and communication and support the optimization of all learning environments (i.e., the acoustic characteristics of school spaces, the hearing abilities of students and teachers, and even the vocal and linguistic dynamics of educators), thus helping to optimize the current neglected hearing conditions of the entire school and its classrooms. The approach is therefore to create effective yet flexible listening, communication, and learning environments that take into account individual differences in students’ hearing and communication access, within the framework of a universal design for learning.

## 5. Conclusions

The A.BA.CO. project’s overarching aim was to review the current challenges and propose priorities for future technological and policy-based interventions. It highlights the importance of inclusive practices, particularly the integration of advanced auditory and communication technologies, to improve educational accessibility for students with hearing difficulties.

The key findings include widespread issues in classroom acoustics, where noise and poor seating arrangements significantly hinder learning. Teachers also face vocal strain, underlining the need for better acoustic design. The project evaluated the performance of assistive technologies like speech-to-text captioning systems, including a prototype developed by A.BA.CO., which showed promise in enhancing communication—but also underscored the necessity for customization due to technological variability.

A key recommendation is the formal integration of educational audiology into school systems, a specialty not yet established in Italy. In countries where it is implemented, educational audiologists play a crucial role in bridging the clinical and educational gaps by designing individualized support programs, managing hearing technologies, and collaborating with teachers and families. Educational audiology is not solely about hearing and hearing health, it is about empowering students to fully participate in auditory and multimodal learning and school communication life, thus supporting the development of each student’s academic potential, social identity, and emotional well-being, within the framework of a universal design for learning

This work lays the groundwork for further contributions and future research to explore to what extent such services can shape the educational paths of students with hearing impairments and support their development as active, empowered members of society.

## Figures and Tables

**Table 1:** A.BA.CO. Part I aimed to promote communicative autonomy and school inclusion for students with hearing loss through a multidisciplinary needs assessment, the development of an AI-based subtitling system, and the creation of a training manual for educators and families. Parts II and III focused on improving accessibility for deaf individuals through LIS (Italian Sign Language) interpretation services and targeted training for public service staff. The gray area outlines the scope of A.BA.CO. Part I, which is described in this article.

Activity	Objective	Coordination	A.BA.CO.
− Multidisciplinary assessment of communication needs in secondary schools (Friuli Venezia Giulia) and universities (University of Perugia) by employing questionnaires. − Design of the AI-based A.BA.CO subtitling system, synchronized with audio/video content and adaptable to all types of hearing loss. − Development of A.BA.CO text, a training manual for school staff and parents, available also in digital format.	Promote communicative autonomy and foster school inclusion by removing barriers to understanding spoken instruction.	Audiology Dept. IRCCS (Istituto di Ricovero e Cura a Carattere Scientifico—Scientific Institute for Research, Hospitalization and Healthcare) Burlo Garofolo, Dr. Eva Orzan	Part I
− Implementation of interpretation and video interpretation services in Italian Sign Language (LIS). − Establishment of a social helpdesk for real-time assistance and linguistic mediation to improve communication and service access. − Training for public administration staff on effective communication with deaf individuals.	− Enhance accessibility for deaf signing individuals through LIS interpretation services. − Raise awareness and improve communication practices in public institutions to foster inclusivity for LIS users.	ENS (Ente Nazionale Sordi—National Deaf Organization) FVG (Friuli Venzia Giulia region), Mrs. Francesca Lisjak	Part II-III

**Table 2:** This table summarizes the content of the eight article produced during the project A.BA.CO.

Title	Main Question	Methodology	Main Outcomes	Key Message
Hearing and listening difficulties in high schools and universities: the results of an exploratory survey of a large number of students and teachers in the Friuli Venezia Giulia and Umbria regions, Italy	How do students and teachers perceive and define their hearing and auditory experiences in the classroom, and what are the listening challenges they face?	Survey: (questionnaire targeting both students and teachers at the University of Perugia and 4 secondary schools in Friuli-Venezia Giulia, Italy, exploring perceived or diagnosed hearing difficulties and the quality of listening in school environments.	8–9% of students reported hearing difficulties, while 27.1% of high school teachers and 12% of university teachers reported hearing challenges. Different key barriers to listening have been reported among high school and university students.	Poor listening environments negatively impact both students and teachers, often without their full awareness, underscoring the need for both improved acoustic conditions and increased understanding of how auditory barriers influence teaching and learning effectiveness.
Teacher’s vocal effort related to environmental noise in schools	What is the relationship between classroom noise levels and teachers’ self-assessed vocal handicap, as measured by the Voice Handicap Index (VHI)?	Pilot exploratory study: Noise level measurements during lessons with “Listen Responsibly” app. Primary school teachers completed the VHI questionnaire after each lesson.	A strong positive correlation (ρ = 0.77) was found between classroom noise and vocal strain. A noise threshold of 59.5 dB was identified as a predictor of increased vocal handicap.	There is a clear link between higher classroom noise and increased vocal strain in teachers, underscoring the urgent need for acoustic improvements and noise management strategies in schools to protect educators’ vocal health.
Extra-auditory effects from noise exposure in classrooms	What are the effects of noise exposure in school environments on students’ learning and health, and on teachers’ vocal strain?	Objective acoustic measurements (in both occupied and unoccupied classrooms) and subjective questionnaires.	Only 3 of 9 classrooms met optimal acoustic standards. Common noise sources included peer conversations, furniture movement, and external traffic. Noise negatively impacted students’ concentration and caused fatigue. Nearly one-third of teachers experienced high vocal effort during a typical two-hour lesson.	School noise significantly affects student learning and teacher vocal health. Acoustic improvements, combined with educational programs to raise awareness, are essential to enhance comfort and reduce strain.
Speech-to-text Captioning and Subtitling in Schools: The Results of a SWOT Analysis	How can speech-to-text captioning systems be strategically implemented in educational settings to support accessibility and inclusion, particularly for students with special needs?	Multidisciplinary strategic analysis (SWOT and TOWS analyses).	Strengths: enhanced communication, inclusivity, and adaptability. Weaknesses: cognitive load, synchronization delays, and varying student needs. Opportunities: innovation, funding, and interdisciplinary collaboration. Threats: poor classroom tech, bad acoustics, and potential social stigma. Final output: strategic recommendations for better usability and customization of the system.	Speech-to-text captioning systems can enhance educational inclusion, and their effective implementation relies on user-centered design and interdisciplinary collaboration within a structured, replicable framework.
Issues and challenges in developing a speech to text system for hearing impaired students—The A.BA.CO. subtitle system story	Can a real-time AI-based speech-to-text system effectively support the communication needs of students with hearing loss in classroom settings?	System design and implementation, evaluation and multidisciplinary feedback from A.BA.CO. project team.	The A.BA.CO. system showed accuracy comparable to leading transcription technologies. Multidisciplinary feedback led to a list of improvements and a roadmap for future development.	The A.BA.CO. project and system mark a significant step toward inclusive education, offering a potentially replicable model for enhancing accessibility in educational environments.
Time delay and frequency analysis of remote microphones	What are the latency and frequency response characteristics of remote microphones used with hearing aids and cochlear implants and how might these affect the effectiveness of the A.BA.CO. speech-to-text system in classroom settings?	Electroacoustic testing, comparison method, and analysis with remote microphones and hearing aids.	Latency differences, comb filter distortion, position and mixing effects were observed.	Optimal performance of the A.BA.CO. system requires customization to account for device-specific delays and prevent audio distortions, ensuring effective communication for hearing aid users.
Lip-Reading Revisited: Advances and Unresolved Questions in a Key Communication Skill	How does lip-reading develop and function across the lifespan, and what is its role in communication, especially in the context of hearing or language impairments and noisy environments?	Literature review: integrative analysis drawing from psycholinguistics, psychophysics, and neurophysiology to explore lip-reading across different developmental stages and contexts.	Lip-reading plays a critical role in speech comprehension—especially in noisy settings or for individuals with hearing or language impairments—and its effectiveness is shaped by visual access, individual abilities, and sociocultural context.	Lip-reading constitutes a lifelong communicative skill that supports speech perception in adverse listening conditions. Its developmental trajectories and individual variability are critical for informing effective intervention strategies.
On the coexistence of captions and sign language as accessibility solutions in educational settings	How do real-time speech-to-text captions and sign language interpreting coexist and contribute to content access for deaf signing students in educational settings?	Empirical literature review focused on experimental studies examining the use of captions and sign language interpretation in education.	Captions support content access for deaf students, even when sign language is also available. The integration of captions and sign language interpreting is feasible. Technological advances (e.g., automatic transcription, AR tools) enhance these accessibility options.	Captions are essential for educational access among deaf signing students and, when integrated with sign language interpreting, align with the universal design for learning to promote inclusive education.

## Data Availability

The original contributions presented in this study are included in the article. Further inquiries can be directed to the corresponding author.
